# The impact of neonatal respiratory distress syndrome on subsequent preschool wheezing in very preterm infants: a multicenter cohort study^☆^

**DOI:** 10.1016/j.clinsp.2026.101023

**Published:** 2026-06-17

**Authors:** Xiaoting Zhang, Yan Li, Xiaoyun Zhong, Lianfang Jing, Mingying Li, Xiangqun Zhao, Yuan Peng, Yuan Shi

**Affiliations:** a Department of Neonatology, Women and Children's Hospital of Chongqing Medical University, Chongqing Health Center for Women and Children, NHC Key Laboratory of Birth Defects and Reproductive Health, Chongqing Municipal Health Commission Key Laboratory of Perinatal Medicine, Chongqing Research Center for Prevention & Control of Maternal and Child Diseases and Public Health, Chongqing, China; b Neonatal Medical Center, Maternity and Child Health Care of Guangxi Zhuang Autonomous Region, Nanning, China; c Department of Neonatology, Children’s Hospital of Chongqing Medical University, National Clinical Research Center for Child Health and Disorders, Ministry of Education Key Laboratory of Child Development and Disorders; China International Science and Technology Cooperation base of Child development and Critical Disorders; Chongqing Key Laboratory of Pediatrics, Chongqing, China

**Keywords:** Respiratory distress syndrome, Wheezing, Very preterm infants, Preschool children, Cohort study

## Abstract

**Background:**

This multicenter cohort study investigated the association between neonatal Respiratory Distress Syndrome (RDS) and wheezing in preschool-aged preterm infants.

**Methods:**

A total of 525 children born at < 32-weeks’ gestation were enrolled from three tertiary hospitals in Southwest China and classified into RDS (n = 275) and non-RDS (n = 250) groups. Current wheezing (≥ 1 parental-reported episode in the past year) outcomes were assessed using the International Study on Asthma and Allergy in Childhood (ISAAC) questionnaire at 3–6 years of age.

**Results:**

The RDS group showed a significantly higher incidence of current wheezing (11.3% vs. 5.6%; aOR = 2.45, 95% CI 1.20–4.98). Subgroup analyses revealed particularly elevated risks among females, infants born at 28⁺⁰–29⁺⁶ weeks, those with birth weights of 1000–1499g, non-IVF conceptions, those with bronchopulmonary dysplasia, and those with < 14-days of oxygen therapy. Sensitivity analyses supported the robustness of these findings.

**Conclusions:**

The study indicates that RDS is an independent risk factor for wheezing in preterm preschoolers and highlights the need for targeted respiratory monitoring in this population.

## Highlights

• Respiratory distress syndrome raises wheezing risk in preterm preschoolers.
• Respiratory distress syndrome preterm group: 11.3% wheezing vs 5.6% others.
• Respiratory distress syndrome is an independent risk for preterm wheezing.
• Targeted respiratory monitoring is needed for these preterm infants.

## Introduction

Preschool wheezing represents a considerable burden on healthcare systems. Emergency department visits for this condition occur at twice the rate, and hospitalizations at five times the rate, of school-aged children with asthma.^1^ Despite its clinical significance, the pathophysiological mechanisms responsible for wheezing remain incompletely understood.^2^

Several mechanisms have been proposed to explain the development of wheezing. First, prematurity has been identified as a potential risk factor. A meta-analysis by Been et al. demonstrated that preterm birth increases the risk of developing asthma by nearly threefold (adjusted Odds Ratio [aOR = 2.81]; 95% CI 2.55 to 3.12).^3^ Second, Bronchopulmonary Dysplasia (BPD), a common complication among preterm infants, has been consistently associated with wheezing episodes.^4^ However, these mechanisms remain subjects of ongoing debate. Some children who develop asthma show no history of BPD.^5^

Respiratory Distress Syndrome (RDS) serves as a leading cause of both early mortality and long-term morbidity in extremely preterm infants, affecting approximately 60% of neonates born before 28-weeks of gestation.^6^ Although advances in surfactant therapy and respiratory support have increased survival rates to 87.6%,^7^ the long-term consequences of these interventions require further investigation, particularly regarding chronic conditions such as airway hyperreactivity, immune dysregulation, and recurrent wheezing.^8^ A previous cohort study involving 448 infants found that RDS significantly increased the incidence of wheezing by 2-years of age (21.0% vs. 8.0%; p < 0.001).^9^ Nevertheless, it remains unclear whether RDS independently contributes to wheezing, and no specific studies have examined its relationship with wheezing in preschool-aged children (3–6 years). This study aims to further investigate the impact of RDS on wheezing in children aged 3–6 years, thereby providing evidence to support prevention strategies targeting preschool wheezing.

## Methods

### Study design, setting and data collection

Participants were recruited through specialized post-discharge follow-up clinics for preterm infants at three tertiary Grade A hospitals (the highest level in China) in Southwest China. Clinical data at the time of Neonatal Intensive Care Unit (NICU) admission were retrospectively extracted from electronic medical records. When the children reached 3–6 years of age, their mothers completed an online questionnaire.

This study utilized the core questionnaire developed by the International Study of Asthma and Allergies in Childhood (ISAAC) Steering Committee,^10^ which is designed to assess the prevalence and related symptoms of asthma, allergic rhinitis, and eczema. In accordance with ISAAC policy, the questionnaire is freely available to non-ISAAC researchers provided that the independence of the study is explicitly stated.^11^ This study was independently conducted and was not part of the ISAAC international collaborative project. To enhance the dataset, the research team supplemented the ISAAC questionnaire with a standardized module to collect retrospectively assessed exposure factors. The study protocol was approved by the institutional ethics committee, and online informed consent was obtained from all participants’ guardians.

### Ethics

The study was conducted following the Declaration of Helsinki and was approved by the Institutional Review Board of Children’s Hospital of Chongqing Medical University (Approval n°2023-18), and registered at www.chictr.org.cn (n° ChiCTR2300074609) (Registration date: 2023-08-10). This study followed the Strengthening of Observational Epidemiological Reporting Study (STROBE) cohort reporting guidelines.

### Study population

This multicenter outpatient cohort study enrolled infants born at < 32-weeks' gestation between 1 June 2017 and 31 March 2022, who were discharged from NICUs and subsequently attended the Preterm Infant Post-Discharge Follow-up Program at outpatient clinics in three tertiary hospitals.

Inclusion required that complete perinatal and NICU electronic medical records be available and participation in scheduled outpatient follow-ups be maintained. Exclusion criteria included: legal guardian refusal; death before age 3-years; loss to follow-up; or major congenital anomalies such as airway malformations (e.g., tracheoesophageal fistula, posterior choanal atresia, cleft palate), diaphragmatic hernia, central nervous system malformations, severe cardiac/renal/gastrointestinal anomalies, and confirmed genetic or metabolic disorders.

### Exposures

Exposure was defined as neonates diagnosed with RDS, compared with non-RDS cases. RDS diagnosis required fulfillment of all criteria within 24 hours post-birth: 1) Clinical signs including respiratory distress with ≥ 2 manifestations (tachypnea > 60 breaths/min, nasal flaring, grunting, or cyanosis and Peripheral Oxygen Saturation [SpO_2_] < 90% in room air); 2) Radiographic confirmation of characteristic chest X-Ray findings (diffuse granular opacities, air bronchograms, or bilateral hypoaeration); 3) Documented symptomatic improvement after surfactant administration or lung recruitment maneuvers.^12^

### Outcomes

The primary outcome was current wheezing, defined by parental report of ≥1 wheezing episode within the preceding 12-months. Current wheezing required fulfillment of ≥1 criterion: 1) Physician-diagnosed asthma with respiratory symptoms in the past year; 2) Use of asthma medications (e.g., inhaled corticosteroids or β₂-agonists) during this period; or 3) The concurrent presence of symptoms and medication use without prior diagnostic confirmation.^13^^,^^14^ Secondary outcomes encompassed incident diseases (diarrhea, influenza, pneumonia, common cold), allergic conditions,^15^ rhinitis according to Allergic Rhinitis and its Impact on Asthma (ARIA) guidelines,^16^ medication utilization patterns, and environmental exposures (tobacco smoke, pet dander) (Supplementary Table S1).

### Covariates and definitions

Variables prespecified for adjustment in multivariable models included: Hypertensive disorders of pregnancy (new-onset systolic blood pressure ≥ 140 mmHg and/or diastolic blood pressure ≥ 90 mmHg after 20-weeks' gestation, confirmed by two measurements ≥ 4 hours apart, with/without proteinuria [≥ 300 mg/24h or albumin-to-creatinine ratio ≥ 30 mg/mmoL] or end-organ dysfunction);^17^ Gestational age (determined by first-trimester crown-rump length [11^+0^-13⁺⁶ weeks]); In Vitro Fertilization (IVF) conception (assisted reproductive technologies including conventional IVF/Intracytoplasmic sperm injection); RDS (Respiratory Distress fulfilling clinical criteria [tachypnea > 60 min, grunting, retractions] plus radiographic diffuse atelectasis, requiring surfactant/mechanical ventilation);^12^ Sepsis (clinically diagnosed neonatal sepsis meeting ≥ 2 systemic inflammatory response criteria [temperature instability, tachycardia > 180 min, leukopenia < 5 × 10⁹/L or leukocytosis > 20 × 10⁹/L, C-Reactive Protein > 10 mg/L, procalcitonin > 2 ng/mL] with/without culture confirmation per Chinese Society of Neonatology guidelines);^18^ Extrauterine Growth Restriction (EUGR; birth weight ≥ 10^th^ percentile declining to < 10^th^ percentile for corrected gestational age at discharge);^19^^,^^20^ Household smoking (cohabitation with ≥ 1 smoker consuming ≥ 1 cigarette/day with ≥ 1 hour/day exposure); Pet exposure (continuous cohabitation with dogs/cats ≥ 3-months) (Supplementary Table S1).

### Sample size calculation

The sample size estimation was calculated by PASS software (2008 v8.0.3). The sample size was determined through dual approaches: Single-group prevalence validation: To detect a clinically relevant deviation from the literature-reported asthma prevalence of 11.7% in very preterm infants (< 32-weeks),^21^ at α = 0.05 (two-sided) with 90% power (δ = 3% margin of error), 486 infants were required. Two-group comparison: Based on pilot data (n = 30) showing a differential prevalence between high-risk (23%) and low-risk (12%) subgroups, a sample size of 246 per group (total n = 492) was calculated to detect this difference at α = 0.05 (two-sided) and 90% power using the two-proportion test. The larger estimate (n = 492) was selected to prioritize testing the hypothesis.

### Statistical analysis

Continuous data were presented as mean ± standard deviation or median with Interquartile Range (IQR), while categorical variables were reported as counts and percentages. Group comparisons utilized Student's *t*-test (normally distributed data), χ^2^ test (expected cell counts > 5), Fisher's exact test (cell counts ≤ 5), or Mann-Whitney U-test (non-normal distributions). Missing data (< 10% across all variables) were handled by mean imputation for continuous variables and mode imputation for categorical variables, following current statistical guidelines for low-level missingness.

Primary analysis employed logistic regression to compute crude Odds Ratios (ORs) with 95% CIs; adjusted ORs were derived from multivariate mixed-effects models incorporating these covariates: in vitro fertilization, gestational hypertension, gestational age, respiratory distress syndrome, Bronchopulmonary Dysplasia (BPD), sepsis, EUGR, household smoking, and pet exposure. Participants in prespecified subgroup analyses were stratified by: 1) Gestational age (< 28-weeks; 28⁺⁰‒29⁺⁶ weeks; 30‒31⁺⁶ weeks); 2) Birth weight (< 1000g; 1000‒1499g; ≥ 1500g); 3) Sex; 4) Assisted conception status; 5) BPD; 6) Oxygen therapy duration (< 14-days; 14‒27 days; ≥ 28-days); 7) Mechanical ventilation exposure. Sensitivity analyses included: Model 1 to 4 (multivariate-adjusted full cohort); Model 5 (propensity score-matched cohort using 1:1 nearest-neighbor matching with caliper = 0.2).

Statistical analyses used SPSS 24.0 (IBM Corp., Armonk, NY) and visualization employed GraphPad Prism 8.0 (GraphPad Software, San Diego, CA). Statistical significance required the 95% CI for adjusted ORs to exclude 1.0.

## Results

### Baseline characteristics of the study population

A total of 525 infants were analyzed (RDS group: n = 275; non-RDS group: n = 250). The median current age was 3.9-years (IQR 3.5–4.2) with no intergroup difference (P = 0.926). Although median gestational age was identical (29.0-weeks), the RDS group showed greater variability (IQR 27.6–30.0 weeks vs. non-RDS 29.0–30.5 weeks; p < 0.001). Birth weight was significantly lower in RDS infants (1200g, IQR 990–1400g vs. non-RDS 1380g, IQR 1260–1600g; p < 0.001). Assisted conception prevalence was higher in the RDS group (27.6% [76/275] vs. 14.8% [37/250]; OR = 2.20, 95% CI 1.42–3.41; p < 0.001). No significant differences existed in sex distribution, delivery mode, singleton pregnancy, antenatal corticosteroid use, or maternal comorbidities (gestational diabetes, chorioamnionitis), except gestational hypertension (14.9% vs. 8.8%; p = 0.031) (Table 1).

**Table 1:** Clinical characteristics of RDS and control groups.

**Clinical characteristics**	**RDS group** **(n = 275), n (%)**	**Control group** **(n = 250), n (%)**	**OR (95% CI)**	**p-value**
**Mother**				
Gestational hypertension	41 (14.9)	22 (8.8)	1.82 (1.05, 3.15)	0.031
Intrahepatic cholestasis of pregnancy	13 (4.7)	15 (6.0)	0.78 (0.36, 1.67)	0.517
Premature rupture of membranes	71 (25.8)	69 (27.6)	0.91 (0.62, 1.35)	0.645
Maternal chorioamnionitis	35 (12.7)	39 (15.6)	0.79 (0.48, 1.29)	0.345
Gestational diabetes mellitus	62 (22.5)	54 (21.6)	1.06 (0.69, 1.59)	0.794
Completed course of antenatal corticosteroids	167 (60.7)	150 (60.0)	1.03 (0.73, 1.46)	0.865
Conceived by in vitro fertilization	76 (27.6)	37 (14.8)	2.20 (1.42, 3.41)	<0.001
Singleton pregnancy	171 (54.5)	143 (45.5)	1.23 (0.87, 1.75)	0.245
**Infants**				
Current age, years, median (IQR)	3.9 (3.0,4.0)	3.9 (3.1,4.2)	‒	0.926
Gestational age, weeks (IQR)	29.0 (27.6, 30.0)	29.0 (29.0, 30.5)	‒	<0.001
Birth weight, grams (IQR)	1200 (990, 1400)	1380 (1260, 1600)	‒	<0.001
Male	156 (56.7)	131 (52.4)	0.84 (0.59, 1.19)	0.320
Vaginal delivery	95 (34.5)	80 (32.0)	1.12 (0.78, 1.61)	0.537
**Hospitalization Indicators**				
Duration of oxygen therapy, days (IQR)	26.9 (6.8,50.7)	4.9 (0,26.0)	-	<0.001
Apgar score at 5 minutes (IQR)	9 (9,9)	9 (9,10)	-	0.004
Endotracheal intubation required	79 (28.7)	21 (8.4)	4.39 (2.61,7.37)	<0.001
Surfactant administration	196 (71.3)	29 (11.6)	18.91 (1.85,30.16)	<0.001
Invasive mechanical ventilation	85 (30.9)	40 (16.0)	2.35 (1.54,3.59)	<0.001
Non-invasive ventilation	264 (96.0)	182 (40.8)	8.97 (4.61,17.43)	<0.001
Extrauterine growth restriction	111 (40.4)	58 (23.2)	2.24 (1.53,3.27)	<0.001
Intraventricular hemorrhage (grade I–IV)	75 (27.3)	56 (22.4)	1.29 (0.87,1.93)	0.198
Necrotizing enterocolitis	36 (13.1)	29 (11.6)	1.15 (0.68,1.94)	0.604
Bronchopulmonary dysplasia	126 (45.8)	58 (23.2)	2.79 (1.92,4.08)	<0.001
Retinopathy of prematurity	115 (41.8)	31 (12.4)	5.08 (3.25,7.93)	<0.001
Periventricular leukomalacia	3 (1.1)	1 (0.4)	2.75 (0.28,26.57)	0.363
Culture-proven sepsis	80 (29.1)	39 (15.6)	2.22 (1.45,3.41)	<0.001

RDS, Respiratory Distress Syndrome; OR, Odds Risk; CI, Confidence Interval; IQR, Interquartile Range.

### Primary outcome

The incidence of the primary endpoint (current wheezing) was significantly higher in the RDS group than in controls (11.3% [31/275] vs. 5.6% [14/250]; OR = 2.01, 95% CI 1.09–3.69; p = 0.020) (Table 2).

**Table 2:** Primary and secondary outcomes.

	**Total (n = 525), n (%)**	**RDS group** **(n = 275), n (%)**	**Control group** **(n = 250), n (%)**	**OR (95% CI)**	**p-value**
**Primary Outcome**					
Current Wheezing	45 (8.6)	31 (11.3)	14 (5.6)	2.01 (1.09, 3.69)	0.020
**Secondary Outcomes**					
**Wheezing Characteristics**					
Frequency of wheezing episodes (past year) 0: (1–2 episodes): (≥ 3 episodes)	483:29:13 (92.0%: 5.5%: 2.5%)	246:18:11 (89.5%: 6.5%: 4.0%)	237:11:2 (94.8%: 4.4%: 0.8%	‒	0.032
Sleep disturbance due to wheezing (per week) 0: (< 1 night): (≥ 1 night)	511:11:3 (97.3%: 2.1%: 0.6%)	265:7:3 (96.4%: 2.5%: 1.1%)	246:4:0 (98.4%: 1.6%: 0)	‒	0.188
Wheeze on exercise	17 (3.2)	8 (2.9)	9 (3.6)	1.25 (0.47, 3.28)	0.655
Nocturnal cough without cold	65 (12.4)	40 (14.5)	25 (10.0)	0.65 (0.38, 1.11)	0.114
Wheezing	82 (15.6)	39 (14.2)	43 (17.2)	1.26 (0.78, 2.01)	0.341
Speech-limiting wheeze (≤1 word per breath)	8 (1.5)	5 (1.8)	3 (1.2)	0.66 (0.16, 2.78)	0.564
Previous physician-diagnosed asthma	71 (13.5)	35 (12.7)	36 (14.4)	0.15 (0.69, 1.90)	0.576
**Rhinitis Characteristics**					
Sneezing (nasal congestion	185 (35.2)	94 (34.2)	91 (36.4)	1.10 (0.77, 1.58)	0.595
Sneezing (nasal congestion (past year)	149 (28.4)	76 (27.6)	73 (29.2)	1.08 (0.74, 1.58)	0.691
Naso-ocular symptoms (itchy eyes with nasal symptoms)	45 (8.6)	18 (6.5)	27 (10.8)	1.73 (0.93, 3.22)	0.082
Nasal symptoms affecting daily activities	40 (7.6)	19 (6.9)	21 (8.4)	1.24 (0.65, 2.36)	0.520
Hay fever	19 (3.6)	10 (3.6)	9 (3.6)	0.99 (0.39, 2.47)	0.982
**Eczema Characteristic**					
Chronic pruritic rash (> 6-months)	40 (7.6)	16 (5.8)	24 (9.6)	1.72 (0.89, 3.32)	0.103
Itchy rash (past year)	30 (5.7)	11 (4.0)	19 (7.6)	1.97 (0.92, 4.23)	0.076
Rash in flexural areas (elbows (knees (ankles)	17 (3.2)	6 (2.2)	11 (4.4)	2.06 (0.75, 5.66)	0.152
Resolved rash	9 (1.7)	6 (2.2)	3 (1.2)	0.55 (0.14, 2.20)	0.387
Previous physician-diagnosed eczema	221 (42.1)	109 (39.6)	112 (44.8)	1.23 (0.87, 1.75)	0.231
**Healthcare Utilization**					
Home oxygen saturation monitoring	116 (22.1)	70 (25.5)	46 (18.4)	0.66 (0.43,1.01)	0.052
Supplemental oxygen therapy	101 (19.2)	57 (20.7)	44 (17.6)	0.82 (0.53,1.26)	0.364
Gastric tube feeding	38 (7.2)	18 (47.4)	20 (8.0)	1.24 (0.64,2.41)	0.521
**Hospitalizations**	169 (32.2)	99 (36.0)	70 (28.0)	0.69 (0.48,1.00)	0.050
Respiratory hospitalization	89 (170)	51 (18.5)	38 (15.2)	0.78 (0.49,1.25)	0.308
ICU admission	15 (2.9)	10 (3.6)	5 (2.0)	0.54 (0.18,1.60)	0.261
RSV-associated hospitalization	62 (11.8)	33 (12.0)	29 (11.6)	0.96 (0.56,1.64)	0.887
**Outpatient visits**	438 (83.4)	228 (82.9)	210 (84.0)	1.08 (0.68,1.72)	0.737
Diarrhea	55 (10.5)	24 (8.7)	31 (12.4)	1.48 (0.84,2.59)	0.170
Influenza	111 (21.1)	56 (20.4)	55 (22.0)	1.10 (0.73,1.68)	0.647
Pneumonia	109 (20.8)	59 (21.5)	50 (20.0)	0.92 (0.60,1.39)	0.682
Asthma	39 (7.4)	23 (8.4)	16 (6.4)	0.75 (0.38,1.45)	0.392
Fever	264 (50.3)	145 (52.7)	119 (47.6)	0.81 (0.58,1.15)	0.241
Common cold	268 (51.0)	143 (52.0)	125 (50.0)	0.92 (0.66,1.30)	0.647
Other conditions	50 (9.5)	19 (6.9)	31 (12.4)	1.91 (1.05, 3.47)	0.032
**Medications**					
Bronchodilators	218 (41.5)	115 (52.8)	103 (41.2)	0.97 (0.68, 1.38)	0.886
Inhaled corticosteroids	245 (46.7)	131 (47.6)	114 (45.6)	0.92 (0.65, 1.29)	0.640
Systemic corticosteroids	85 (16.2)	51 (18.5)	34 (13.6)	0.69 (0.43, 1.11)	0.124
Leukotriene receptor antagonists	7 (1.3)	1 (0.4)	6 (2.4)	6.74 (0.81, 56.35)	0.042
Antihistamines	155 (29.5)	74 (26.9)	81 (32.4)	1.30 (0.89, 1.89)	0.168
Allergen immunotherapy	11 (2.1)	6 (2.2)	5 (2.0)	0.91 (0.27, 3.03)	0.884
Antibiotics	329 (62.7)	171 (62.2)	158 (63.2)	1.04 (0.73, 1.49)	0.810
Traditional Chinese medicine	98 (18.7)	53 (19.3)	45 (18.0)	0.92 (0.59, 1.43)	0.709
No medication use	90 (17.1)	54 (19.6)	36 (14.4)	0.69 (0.43, 1.09)	0.112
**Allergy History**					
Drug allergy	109 (20.8)	60 (21.8)	49 (19.6)	0.87 (0.57, 1.33)	0.531
Penicillin allergy	6 (1.1)	5 (1.8)	1 (0.4)	0.22 (0.03, 1.87)	0.219
Cephalosporin allergy	8 (1.5)	7 (2.5)	1 (0.4)	0.15 (0.02, 1.26)	0.071
Other drug allergies	15 (2.9)	6 (2.2)	9 (3.6)	1.67 (0.59, 4.77)	0.330
**Environmental Exposures**					
Household tobacco smoke exposure	263 (50.1)	148 (53.8)	115 (46.0)	0.73 (0.51, 1.03)	0.074
Daily smoking in household	194 (37.0)	107 (38.9)	87 (34.8)	0.84 (0.59, 1.19)	0.330
Exposure to dogs or cats	63 (12.0)	40 (14.5)	23 (9.2)	0.59 (0.35, 1.03)	0.060

RDS, Respiratory Distress Syndrome; OR, Odds Risk; CI, Confidence Interval; IQR, Interquartile Range; ICU, Intensive Care Unit; RSV, Respiratory Syncytial Virus.

### Secondary outcomes

Wheezing frequency analysis revealed group differences (p = 0.032), with higher rates of recurrent wheezing (≥ 3 episodes/ past year) in RDS infants (4.0% vs. 0.8%). However, no significant differences were observed for sleep-disrupting wheezing (p = 0.188), exercise-induced wheezing (OR = 1.25; 95% CI 0.48–3.25; p = 0.655), non-cold nocturnal cough (OR = 0.65; 95% CI 0.38–1.11; p = 0.114), speech-restricted attacks (OR = 0.66; 95% CI 0.16–2.78; p = 0.564), or prior asthma diagnosis (OR = 0.15; 95% CI 0.69–1.90; p = 0.576). As shown in Table 2, the following non-respiratory outcomes demonstrated no statistically significant differences: rhinorrhea, eczema prevalence (assessed using UK Working Party criteria), healthcare utilization (emergency visits or hospitalizations), medication use (bronchodilators or corticosteroids), allergic history, and environmental exposures (tobacco smoke or pet ownership).

### Subgroup analysis

**Fig. 1:**
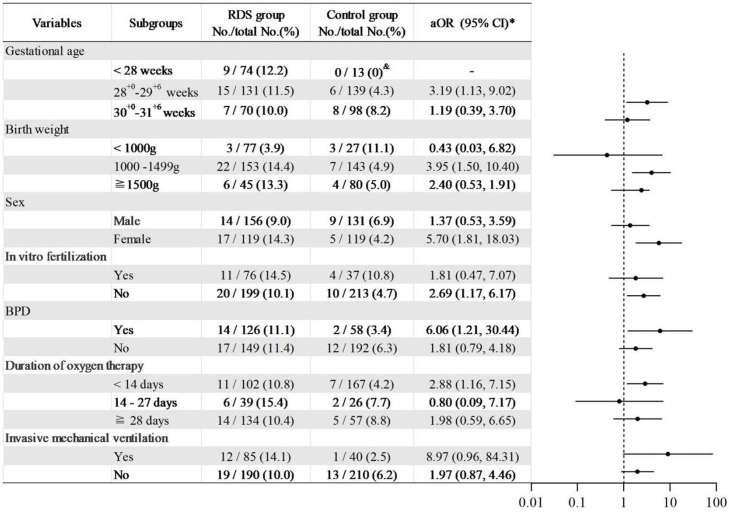
Subgroup analysis of Current Wheezing on RDS and Control groups RDS, Respiratory Distress Syndrome; OR, Odds Risk; CI, Confidence Interval; IQR, Interquartile Range; BPD, Bronchopulmonary Dysplasia; EUGR, Extrauterine Growth Restriction. * Adjusted for in vitro fertilization, gestational hypertension, gestational age, RDS, BPD, sepsis, EUGR, household tobacco smoke exposure and pet exposure. ^&^ No wheezing events. The data represent only the survivors with follow-up records.

In stratified analyses, multivariable-adjusted models revealed significantly elevated current wheezing risk in specific subgroups: gestational age 28⁺⁰‒29⁺⁶ weeks (Aor = 3.19, 95% CI 1.13–9.02; p = 0.028), birth weight 1000–1499g (aOR = 3.95, 95% CI 1.50–10.40; p = 0.005), females (aOR = 5.70, 95% CI 1.81–18.03; p = 0.003), non-IVF conception (aOR = 2.69, 95% CI 1.17–6.17; p = 0.020), BPD diagnosis (aOR = 6.06, 95% CI 1.21–30.44; p = 0.029), and oxygen therapy < 14-days (aOR = 2.88, 95% CI 1.16–7.15; p = 0.023) (Fig. 1 and Supplementary Table S2).

### Sensitivity analysis

In sensitivity analyses examining the association between RDS and current wheezing, the authors observed consistent and statistically significant results across multiple modeling approaches. After adjustment for potential confounders, RDS remained significantly associated with current wheezing: Model 1 (adjusted for in vitro fertilization, gestational hypertension, gestational age, RDS, BPD, sepsis, and EUGR) showed an adjusted Odds Ratio (aOR) of 2.55 (95% CI 1.26‒5.17; p = 0.009); Model 2 (additional adjustment for household tobacco smoke exposure) yielded an aOR of 2.52 (95% CI 1.24‒5.11; p = 0.011); Model 3 (additional adjustment for pet exposure) demonstrated an aOR of 2.47 (95% CI 1.21‒5.03; p = 0.013); and Model 4 (adjusted for all above covariates) maintained the association (aOR = 2.45; 95% CI 1.20‒4.98; p = 0.014). In the propensity score-matched cohort (Model 5), where 189 RDS cases were matched 1:1 with controls using nearest-neighbor matching (caliper = 0.2), the association persisted with an aOR of 2.52 (95% CI 1.10‒5.78; p = 0.029). Model 6 was adjusted for Model 1 plus the duration of oxygen therapy, yielding an aOR of 2.54 (95% CI 1.24‒5.19; p = 0.011). Model 7 built upon Model 4 by further adjusting for the duration of oxygen therapy, resulting in an aOR of 2.43 (95% CI 1.18‒5.00, p = 0.016). Model 8 extended Model 5 by adding the duration of oxygen therapy, with an aOR of 2.51 (95% CI 1.09‒5.82, p = 0.031) (Table 3).

**Table 3:** Sensitive analysis of Current Wheezing on RDS and Control groups.

**Model**	**RDS group, n (%)**	**Control group, n (%)**	**aOR (95% CI)**	**p-value**
Model 1^a^	31 (11.3)	14 (5.6)	2.55 (1.26‒5.17)	0.009
Model 2^b^	31 (11.3)	14 (5.6)	2.52 (1.24‒5.11)	0.011
Model 3^c^	31 (11.3)	14 (5.6)	2.47 (1.21, 5.03)	0.013
Model 4^d^	31 (11.3)	14 (5.6)	2.45 (1.20, 4.98)	0.014
Model 5^e^	21 (11.1)	9 (4.7)	2.52 (1.10, 5.78)	0.029
Model 6^f^	31 (11.3)	14 (5.6)	2.54 (1.24, 5.19)	0.011
Model 7^g^	31 (11.3)	14 (5.6)	2.43 (1.18, 5.00)	0.016
Model 8^h^	21 (11.1)	9 (4.7)	2.51 (1.09, 5.82)	0.031

RDS, Respiratory Distress Syndrome; OR, Odds Risk; CI, Confidence Interval; IQR, Interquartile Range; BPD, Bronchopulmonary Dysplasia; EUGR, Extrauterine Growth Restriction

a Model 1 adjusted for in vitro fertilization, gestational hypertension, gestational age, RDS, BPD, sepsis, EUGR.

b Model 2 adjusted for Model 1 plus household tobacco smoke exposure.

c Model 3 adjusted for Model 1 plus pet exposure.

d Model 4 adjusted for Model 1 plus household tobacco smoke exposure and pet exposure.

e Model 5: propensity score-matched cohort using 1:1 nearest-neighbor matching with caliper = 0.2, and used the same confounding factors with Model 4 (The matched post-coefficient balance table was shown in Supplementary Table S3.

f Model 6 adjusted for Model 1 plus duration of oxygen therapy.

g Model 7 adjusted for Model 4 plus duration of oxygen therapy.

h Model 8 adjusted for Model 5 plus duration of oxygen therapy.

## Discussion

This study demonstrates that RDS significantly increases preschool wheezing risk in very preterm infants (< 32-weeks' gestation), with observed incidence of 11.3% in the RDS group versus 5.6% in non-RDS infants (OR = 2.01, 95% CI 1.09–3.69; p = 0.020). Results remained consistent after multivariate adjustment for confounders, including gestational age and BPD (aOR = 2.45, 95% CI 1.20–4.98; p = 0.014). Verification in a propensity score-matched cohort showed significantly higher wheezing incidence among infants with RDS compared to controls (11.1% vs. 4.7%; aOR = 2.52, 95% CI 1.10–5.78; p = 0.029).

These findings align with previous studies: Koivisto et al.'s cohort of 448 infants demonstrated that RDS increased wheezing incidence within the first two years of life (47/224 vs. 18/224, p < 0.005),^22^ and Cha et al.'s national cohort study of 2,224,476 infants found that RDS elevated risks of asthma (OR = 1.09, 95% CI 1.05–1.14), early-onset asthma (< 2-years; OR = 1.16, 95% CI 1.06–1.26), and severe asthma (OR = 1.11, 95% CI 0.98–1.27).^23^ However, earlier research had not specifically examined preschoolers, and this study is the first to identify a significant association between RDS and preschool wheezing in very preterm infants using an outpatient cohort design.

Subgroup analyses identified elevated wheezing risk in specific populations: gestational age 28^+0^ to 29⁺⁶ weeks (aOR = 3.19, 95% CI 1.13–9.02; p = 0.028), birth weight 1000–1499g (aOR = 3.95, 95% CI 1.50–10.40; p = 0.005), presence of BPD (aOR = 6.06, 95% CI 1.21–30.44; p = 0.029), and duration of oxygen therapy less than two weeks (aOR = 2.88, 95% CI 1.16–7.15; p = 0.023).

The mechanisms underlying high wheezing risk in very preterm infants remain incompletely understood.^24^ Numerous studies have investigated associations between gestational age and childhood wheezing disorders, indicating preterm birth correlates with increased wheezing risk.^5^^,^^25^ Meta-analyses by Been et al. showed very preterm infants (< 32-weeks) had elevated asthma risk (unadjusted OR = 3.00, 95% CI 2.61–3.44; aOR = 2.81, 95% CI 2.55–3.12).^3^ Der Voort et al.'s meta-analysis of 147,252 children from 31 cohort studies found preterm birth associated with increased preschool wheezing (pooled OR = 1.34, 95% CI 1.25–1.43) and school-age asthma (pooled OR = 1.40, 95% CI 1.18–1.67).^26^ Recent literature highlights that prenatal and perinatal factors, including infections, oxygen therapy, and mechanical ventilation, adversely impact lung development and airway responsiveness.^3^ RDS represents the most common perinatal acute lung injury; its pro-inflammatory cytokines (e.g., Tumor Necrosis Factor-alpha [TNF-α] and Interleukin-6 [IL-6]) induce bronchial hyperresponsiveness, while hyperoxia alters pulmonary morphogenesis. Collectively, these mechanisms contribute to elevated asthma incidence.^27^

Animal models demonstrate intermittent hypoxia (cycling between 50% and 10% oxygen) increases airway acetylcholine sensitivity and upregulates inducible nitric oxide synthase.^28^ Clinically, cumulative oxygen exposure during postnatal days 1–3 correlates with subsequent asthma medication use.^29^ Although BPD correlates with wheezing incidence,^4^^,^^30^ its predictive value remains incomplete, 40% of non-BPD infants develop significant respiratory disease.^5^^,^^31^ Thus, RDS-associated inflammation may independently increase wheezing risk through pathways distinct from BPD progression. The outpatient cohort provides novel evidence that RDS independently elevates preschool wheezing risk in very preterm infants after accounting for perinatal confounders.

### Strengths and limitations

This study utilized a large-scale outpatient cohort, validated through propensity score-matched analysis while rigorously controlling for key confounders, including Bronchopulmonary Dysplasia (BPD) and gestational age, thereby substantially strengthening causal inferences. The authors employed the self-administered version of the ISAAC questionnaire, which may introduce recall bias. A key limitation of this study is survivorship bias. As a result, the findings can only be generalized to infants who survived and remained in the study without dropping out. They should not be applied to critically ill infants who either died or were lost to follow-up.

## Conclusions

This study demonstrates that RDS in extremely preterm infants significantly increases the risk of current wheezing at preschool age. Future studies must validate targeted interventions in high-risk populations ‒ particularly those experiencing loss to follow-up ‒ through enhanced retention strategies such as mobile health monitoring and community-based outreach programs to ensure comprehensive outcome assessment.

## Abbreviations

RDS, Respiratory Distress Syndrome; IVF, In Vitro Fertilization; BPD, Bronchopulmonary Dysplasia; NICU, Neonatal Intensive Care Unit; ISAAC, International Study on Asthma and Allergy in Childhood; EUGR, Extrauterine Growth Restriction.

## Data availability statement

The datasets generated during and/or analyzed during the current study are available from the corresponding author on reasonable request.

## Transparency statement

The lead author (the manuscript’s guarantor) affirms that the manuscript is an honest, accurate, and transparent account of the study being reported; that no important aspects of the study have been omitted; and that any discrepancies from the study as planned (and, if relevant, registered) have been explained.

## Patient and Public Involvement statement

Not applicable.

## Authors' contributions

Dr XT.Z contributed to the collection of data, drafted the initial manuscript and reviewed the manuscript; Dr MY.L, Dr XQ.Z, Dr LF.J and Dr Y.P contributed to the collection of data, Dr Y.L and Dr XY.Z contributed to the medical outcome assessors; Dr Y.S conceptualized and designed the study, funding acquisition, and critically reviewed the manuscript for important intellectual content. All authors revised the manuscript and approved the final manuscript as submitted. All authors had full access to the data in the present study and accept responsibility for submitting for publication.

## Funding

1. Technology innovation and application development of Chongqing, China (cstc2019jscx‐msxmX0232).
2. National Key Research and Development Program of China (2022YFC2704803).
3. National Key Clinical Specialty Construction Project (Neonatology), 2024.

## Declaration of competing interest

The authors declare no conflicts of interest.

## Appendix Supplementary materials

Image, application 1

## Footnotes

Supplementary material associated with this article can be found, in the online version, at doi:10.1016/j.clinsp.2026.101023.
