# Supplementary material for: The impact of neonatal respiratory distress syndrome on subsequent preschool wheezing in very preterm infants: a multicenter cohort study
Source: Clinics (Sao Paulo). 2026 Jun 17;81:101023. doi: 10.1016/j.clinsp.2026.101023 (PMC13293732; doi:10.1016/j.clinsp.2026.101023)
Supplement: Supplementary file 1 [file mmc1.docx]

CLINICS-D-25-01600_Supplementary Material

**Supplementary Table S1** Variables and definitions.

| Variables | **Definitions** |
| --- | --- |
| **Primary Outcomes** |  |
| Current wheeze | Parental report of ≥1 wheezing episode within the preceding 12-months |
|  | Fulfillment of ≥1 criterion: 1) Physician-diagnosed asthma with respiratory symptoms in past year; 2) Asthma medication use (inhaled corticosteroids/β2-agonists); 3) Concurrent symptoms and medication use without prior diagnosis recall. |
| **Secondary Outcomes** |  |
| Incident respiratory diseases | New-onset events of diarrhea, influenza, pneumonia, common cold |
| **Diarrhea** | ≥3 loose or watery stools per 24 hours |
| **Influenza** | Acute fever (≥38°C) with cough/sore throat/myalgia + laboratory confirmation |
| **Pneumonia** | New infiltrate on chest radiograph + ≥2 of: |
|  | - Fever >38.5°C |
|  | - Tachypnea (RR: infant >50 min; 1‒5yr >40 min) |
|  | - Crackles on auscultation |
|  | - Oxygen saturation <90% (room air) |
| **Common cold** | Rhinorrhea/nasal congestion/sneezing + no or low-grade fever (<38°C) + absence of lower respiratory symptoms |
| Eczema | Diagnosed per UK Working Party criteria [ref] (pruritus + ≥3 minor criteria: history of flexural dermatitis, etc.) |
| Rhinitis | Defined per ARIA guidelines [ref] (symptoms ≥4-days/week and >4-weeks) |
| Medication utilization | Patterns of bronchodilator/corticosteroid use, including **Corticosteroids (ICS), Long-Acting β2-Agonists (LABA), Short-acting β2-agonists (SABA) and Leukotriene Receptor Antagonists (LTRA).** |
| Environmental exposures | Documented exposure to tobacco smoke or pet dander |
| Household smoking | Cohabitation with ≥1 smoker consuming ≥1 cigarette/day with ≥1 hour/day exposure |
| Pet exposure | Continuous cohabitation with dogs/cats ≥3-months |
| **Covariates** |  |
| Hypertensive disorders of pregnancy | New-onset systolic BP ≥140 mmHg and/or diastolic BP ≥90 mmHg after 20-weeks’ gestation (confirmed by ≥2 measurements ≥4-hours apart), with/without proteinuria (≥300 mg/24h or ACR ≥30 mg/mmoL) or end-organ dysfunction |
| Gestational age | Determined by first-trimester fetal crown-rump length measurement at 11-13⁺⁶ weeks |
| IVF conception | Conception through assisted reproductive technologies (conventional IVF or ICSI) |
| RDS | Respiratory distress with tachypnea >60/min, grunting, retractions; plus, radiographic diffuse atelectasis; requiring surfactant/mechanical ventilation |
| Sepsis | Meeting ≥2 criteria: temperature instability, tachycardia >180 min, leukopenia <5×10⁹/L or leukocytosis >20×10⁹/L, CRP >10 mg/L, PCT >2 ng/mL; with/without culture confirmation per Chinese guidelines |
| EUGR | Birth weight ≥10^th^ percentile declining to <10^th^ percentile for corrected gestational age at discharge |
| Household smoking | Cohabitation with ≥1 smoker consuming ≥1 cigarette/day with ≥1 hour/day exposure |
| Pet exposure | Continuous cohabitation with dogs/cats ≥3-months |

IVF, In Vitro Fertilization; ICSI, Intracytoplasmic Sperm Injection; RDS, **Respiratory Distress Syndrome;** EUGR, Extrauterine Growth Restriction

**Supplementary Table S2** Subgroup analysis of Current Wheezing on RDS and Control groups.

| **Variables** | **Subgroups** | **Total Nº / Total n (%)** | **RDS group Nº / Total n. (%)** | **Control group Nº / Total n (%)** | **OR (95% CI)** | **p-value** | **aOR (95% CI)*** | **p-value** |
| --- | --- | --- | --- | --- | --- | --- | --- | --- |
| Gestational age | < 28 weeks | 9 / 87 (10.3) | 9 / 74 (12.2) | 0 / 13 (0)^&^ | 1.14 (1.05, 1.24) | 0.344 | ‒ | ‒ |
|  | 28^+0^-29^+6^ weeks | 21 / 270 (7.8) | 15 / 131 (11.5) | 6 / 139 (4.3) | 2.87 (1.08, 7.63) | 0.029 | 3.19 (1.13, 9.02) | **0.028** |
|  | ≥ 30^+0^ weeks | 15/ 168 (8.9) | 7 / 70 (10.0) | 8 / 98 (8.2) | 1.02 (0.93, 1.13) | 0.681 | 1.19 (0.39, 3.70) | 0.760 |
| Birth weight | < 1000g | 6 / 104 (5.8) | 3 / 77 (3.9) | 3 / 27 (11.1) | 0.32 (0.06, 1.72) | 0.366 | 0.43 (0.03, 6.82) | 0.547 |
|  | 1000‒1499g | 29 / 296 (9.8) | 22 / 153 (14.4) | 7 / 143 (4.9) | 3.26 (1.35, 7.89) | 0.006 | 3.95 (1.50, 10.40) | **0.005** |
|  | ≥ 1500g | 10 / 125 (8.0) | 6 / 45 (13.3) | 4 / 80 (5.0) | 2.92 (0.78, 10.97) | 0.192 | 2.40 (0.53, 1.91) | 0.258 |
| Sex | Male | 23 / 287 (8.0) | 14 / 156 (9.0) | 9 / 131 (6.9) | 1.34 (0.56, 3.19) | 0.513 | 1.37 (0.53, 3.59) | 0.518 |
|  | Female | 22 / 238 (9.2) | 17 / 119 (14.3) | 5 / 119 (4.2) | 3.80 (1.35, 10.67) | 0.007 | 5.70 (1.81, 18.03) | **0.003** |
| In vitro fertilization | Yes | 15 / 113 (13.3) | 11 / 76 (14.5) | 4 / 37 (10.8) | 1.39 (0.41, 4.72) | 0.590 | 1.81 (0.47, 7.07) | 0.391 |
|  | No | 30 / 412 (7.3) | 20 / 199 (10.1) | 10 / 213 (4.7) | 2.27 (1.03, 4.97) | 0.037 | 2.69 (1.17, 6.17) | **0.020** |
| BPD | Yes | 16 / 184 (8.7) | 14 / 126 (11.1) | 2 / 58 (3.4) | 3.50 (0.77, 15.94) | 0.087 | 6.06 (1.21, 30.44) | **0.029** |
|  | No | 29 / 341 (8.5) | 17 / 149 (11.4) | 12 / 192 (6.3) | 1.93 (0.89, 4.18) | 0.090 | 1.81 (0.79, 4.18) | 0.162 |
| Duration of oxygen therapy | < 14 days | 18 / 269 (6.7) | 11 / 102 (10.8) | 7 / 167 (4.2) | 2.76 (1.04, 7.38) | 0.036 | 2.88 (1.16, 7.15) | **0.023** |
|  | 14‒27 days | 8 / 65 (12.3) | 6 / 39 (15.4) | 2 / 26 (7.7) | 2.18 (0.41, 11.76) | 0.355 | 0.80 (0.09, 7.17) | 0.840 |
|  | ≥ 28 days | 19 / 191 (9.9) | 14 / 134 (10.4) | 5 / 57 (8.8) | 1.21 (0.42, 3.54) | 0.723 | 1.98 (0.59, 6.65) | 0.270 |
| Invasive mechanical ventilation | Yes | 13 / 125 (10.4) | 12 / 85 (14.1) | 1 / 40 (2.5) | 6.41 (0.80, 51.15) | 0.047 | 8.97 (0.96, 84.31) | 0.055 |
|  | No | 32 / 400 (8.0) | 19 / 190 (10.0) | 13 / 210 (6.2) | 1.68 (0.81, 3.50) | 0.161 | 1.97 (0.87, 4.46) | 0.103 |

RDS, **Respiratory Distress Syndrome;** OR, Odds Risk; CI, Confidence Interval; BPD, Bronchopulmonary Dysplasia.

^&^ No wheezing events. The data represent only the survivors with follow-up records.

* Adjusted for in vitro fertilization, gestational hypertension, gestational age, RDS, BPD, sepsis, EUGR, household tobacco smoke exposure and pet exposure.

**Supplementary Table S3** Propensity score matching results: RDS vs. control groups.

|  | **Total, n (%)**  **(n = 380)** | **RDS group, n (%)**  **(n = 190)** | **Control group, n (%) (n = 190)** | **95% CI** | **p-value** |
| --- | --- | --- | --- | --- | --- |
| **Current age**, years, median (IQR) | 4.0 (3.0, 5.0) | 4.0 (3.1, 5.0) | 4.0 (3.0, 5.0) | ‒ | 0.840 |
| Gestational age, weeks (IQR) | 29.0 (29.0, 30.3) | 29.2 (29.0.30.3) | 29.0 (29.0, 30.3) | ‒ | 0.434 |
| Birth weight, grams (IQR) | 1320 (1150, 1490) | 1305 (1150, 1490) | 1320 (1150, 1513) | ‒ | 0.434 |
| Male | 204 (53.7) | 104 (54.7) | 100 (52.6) | 1.09(0.73, 1.63) | 0.681 |
| Vaginal delivery | 121 (31.8) | 58 (30.5) | 63 (33.2) | 0.89(0.58, 1.36) | 0.582 |
| Singleton pregnancy | 228 (60.0) | 122 (64.2) | 106 (55.8) | 1.42(0.94, 2.15) | 0.094 |
| In vitro fertilization | 72 (18.9) | 40 (21.1) | 32 (16.8) | 1.32(0.78, 2.20) | 0.295 |
| Completed course of antenatal corticosteroids | 206 (54.2) | 106 (55.8) | 100 (52.6) | 1.14(0.76, 1.70) | 0.537 |
| Gestational hypertension | 45 (11.8) | 25 (13.2) | 20 (10.5) | 1.29(0.69, 2.41) | 0.427 |
| Intrahepatic cholestasis of pregnancy | 24 (6.3) | 11 (5.8) | 13 (6.8) | 0.84(0.37, 1.92) | 0.673 |
| Premature rupture of membranes | 93 (24.5) | 52 (27.4) | 41 (21.6) | 1.37(0.86, 2.19) | 0.189 |
| Maternal chorioamnionitis | 30 (7.9) | 16 (8.4) | 14 (7.4) | 1.16(0.55, 2.44) | 0.704 |
| Gestational diabetes mellitus | 76 (20.0) | 43 (22.6) | 33 (17.4) | 1.39(0.84, 2.31) | 0.200 |

RDS, **Respiratory Distress Syndrome;** OR, Odds Risk; CI, Confidence Interval; IQR, Interquartile Range.
